# CHASE domain-containing receptors play an essential role in the cytokinin response of the moss *Physcomitrella patens*

**DOI:** 10.1093/jxb/erv479

**Published:** 2015-11-23

**Authors:** Klaus von Schwartzenberg, Ann-Cathrin Lindner, Njuscha Gruhn, Jan Šimura, Ondřej Novák, Miroslav Strnad, Martine Gonneau, Fabien Nogué, Alexander Heyl

**Affiliations:** ^1^Biozentrum Klein Flottbek, Universität Hamburg, Ohnhorststr. 18, D-22609 Hamburg, Germany; ^2^Institute for Biology/ Applied Genetics, Dahlem Centre of Plant Sciences, Freie Universität Berlin, Albrecht-Thaer-Weg 6, D-14195 Berlin, Germany; ^3^Laboratory of Growth Regulators & Department of Chemical Biology and Genetics, Centre of the Region Haná for Biotechnological and Agricultural Research, Palacký University & Institute of Experimental Botany ASCR, Šlechtitelů 27, CZ-783 71 Olomouc, Czech Republic; ^4^Institut Jean-Pierre Bourgin, UMR1318 INRA-AgroParisTech, INRA Centre de Versailles-Grignon, Route de St-Cyr, 78026 Versailles Cedex, France; ^5^ Biology Department, Adelphi University, Science 116, 1 South Avenue, PO Box 701, Garden City, NY 11530-070, USA

**Keywords:** Bryophyte, cytokinin, cytokinin receptor, evolution, moss, *Physcomitrella patens*, phytohormone, plant growth regulator, signaling, two-component system.

## Abstract

While the molecular basis for cytokinin action is quite well understood in flowering plants, little is known about the cytokinin signal transduction in early diverging land plants. The genome of the bryophyte *Physcomitrella patens* (Hedw.) B.S. encodes three classical cytokinin receptors, the CHASE domain-containing histidine kinases, CHK1, CHK2, and CHK3. In a complementation assay with protoplasts of receptor-deficient *Arabidopsis thaliana* as well as in cytokinin binding assays, we found evidence that CHK1 and CHK2 receptors can function in cytokinin perception. Using gene targeting, we generated a collection of *CHK* knockout mutants comprising single (Δ*chk1*, Δ*chk2*, Δ*chk3*), double (Δ*chk1,2*, Δ*chk1,3*, Δ*chk2,3*), and triple (Δ*chk1,2,3*) mutants. Mutants were characterized for their cytokinin response and differentiation capacities. While the wild type did not grow on high doses of cytokinin (1 µM benzyladenine), the Δ*chk1,2,3* mutant exhibited normal protonema growth. Bud induction assays showed that all three cytokinin receptors contribute to the triggering of budding, albeit to different extents. Furthermore, while the triple mutant showed no response in this bioassay, the remaining mutants displayed budding responses in a diverse manner to different types and concentrations of cytokinins. Determination of cytokinin levels in mutants showed no drastic changes for any of the cytokinins; thus, in contrast to Arabidopsis, revealing only small impacts of cytokinin signaling on homeostasis. In summary, our study provides a first insight into the molecular action of cytokinin in an early diverging land plant and demonstrates that CHK receptors play an essential role in bud induction and gametophore development.

## Highlight

Functional characterization of the three classical CHASE domain-containing receptors from *Physcomitrella patens* reveals their key role in the moss cytokinin response.

## Introduction

Phytohormones regulate many processes in plants such as the development of tissues and organs and the response to changes in the environment. One class of phytohormones, the cytokinins, is comprised of adenine derivatives carrying an isoprenoid or an aromatic side chain at the *N* ^*6*^-position (Mok and Mok, 2001). Cytokinin signaling is mediated via a multistep His-to-Asp phosphorelay system, a variant of the bacterial two-component system (TCS). While this type of signaling system is widespread in prokaryotes, it is unique to plants among multicellular eukaryotes (Heyl and Schmülling, 2003). For *Arabidopsis thaliana*, the current model of this signaling pathway predicts that the cytokinin ligand is bound by hybrid histidine kinase receptors via the cyclases/histidine kinases associated sensory extracellular (CHASE) domain (Anantharaman and Aravind, 2001; Mougel and Zhulin, 2001; Heyl *et al.*, 2007). These CHASE domain-containing histidine kinases (CHKs) were shown to localize mainly to the endoplasmic reticulum (ER) (Caesar *et al.*, 2011; Lomin *et al.*, 2011; Wulfetange *et al.*, 2011). The binding of the cytokinin ligand causes an autophosphorylation of the CHK receptor. After an intramolecular phosphotransfer, the signal is transmitted by phosphorylation to histidine phosphotransmitter proteins (HPTs), which shuttle between the cytoplasm and the nucleus (Punwani *et al.*, 2010). In the nucleus, the HPTs activate type-B response regulators (RRBs), transcriptional regulators belonging to the class of Myb transcription factors via phosphorylation. Subsequently these transcription factors initiate the transcription of their target genes, one group of which are the type-A RRs (RRAs). RRA proteins have been shown to be involved in a negative feedback mechanism of the cytokinin signaling pathway (Hwang and Sheen, 2001; To *et al.*, 2004). Most of the research on this signaling pathway has been done using the model plant Arabidopsis, but work in other plants species also contributed to the elucidation of the functioning of the pathway (Heyl *et al.*, 2006a; Hellmann *et al.*, 2010).

One of the open questions in cytokinin biology is the origin and evolution of this regulatory system and its contribution to the conquest of land by plants. Nevertheless, our knowledge of the cytokinin biology of algae and early diverging land plants is very limited (Tarakhovskaya *et al.*, 2007; Pils and Heyl, 2009; von Schwartzenberg, 2009; Frebort *et al.*, 2011; Spichal, 2012; Gruhn and Heyl, 2013). The streptophyta alga *Klebsormidium flaccidum* was recently shown to code for all parts of the TCS system in the evolution of the green lineage prior to the conquest of land (Hori *et al.*, 2014). The moss *Physcomitrella patens* as an early divirging land plant also encodes all protein families involved in cytokinin biosynthesis, metabolism, and signaling (Pils and Heyl, 2009; Frébort *et al.*, 2011; Spíchal, 2012; Gruhn and Heyl, 2013; Gruhn *et al.*, 2014). Due to its simple developmental differentiation and its responsiveness to several plant hormones, *P. patens* is a long-standing model regarding hormonal action and homeostasis (Wang *et al.*, 1981; Cove, 2005; Decker *et al.*, 2006; von Schwartzenberg, 2006, 2009). Twenty different endogenous cytokinins were detected and quantified in *P. patens*, and the generation of cytokinin-deficient plants revealed the importance of extracellular cytokinins for bud formation (von Schwartzenberg *et al.*, 2007). Furthermore, the apparent absence of adenylate isopentenyltransferases (IPTs), the key enzymes for cytokinin production in flowering plants, makes *P. patens* an interesting organism for studying cytokinin biology in general (Yevdakova and von Schwartzenberg, 2007; Yevdakova *et al.*, 2008; Frébort *et al.*, 2011; Patil and Nicander, 2013; Lindner *et al.*, 2014). While cytokinin metabolism has a long tradition as a topic in *P. patens* research (reviewed by von Schwartzenberg, 2009), the signaling of this phytohormone has only recently attracted the attention of researchers (Pils and Heyl, 2009; Ishida *et al.*, 2010). Last year a new subfamily of cytokinin receptors was described containing eight members from *P. patens* (Gruhn *et al.*, 2014). This discovery makes this moss the only plant which encodes both classical and newly identified cytokinin receptors in its genome, and it raises the question of the biological role of both receptor subfamilies in *P. patens*. 

Here we present the characterization of the three classical CHASE domain-containing histidine kinase cytokinin receptors from *P. patens.* Following the suggested nomenclature (Heyl *et al.*, 2013), we refer to them as CHK1, CHK2, and CHK3 and describe their role in differentiation processes of the moss. Our results show that the proteins can function as cytokinin receptors in different assays, and analysis of single, double, and the triple mutants demonstrated that CHK1, CHK2, and CHK3 are necessary for cytokinin perception by the moss. The results highlight the importance of these receptors for the cytokinin response in this early diverging land plant species.

## Materials and methods

### CHK *full-length cDNAs*

For the functional assays it was essential to isolate the respective cDNA clone for each of the three receptor genes (genomic loci: *CHK1*, Pp1s50_141; *CHK2*, Pp1s194_72; *CHK3*, Pp1s252_49; see http://www.cosmoss.org; Lang *et al.*, 2005). By using degenerated *AHK4* primers (degAHK4 for, gcnathgaycargaracnttygc; and deg*AHK4* rev, tgngcngtytgngcrtartc) on wild-type protonemal cDNA, two 942bp fragments (*CHK1*–942 and *CHK2*–942) were amplified, subcloned, and sequenced. To retrieve the *CHK1* sequence (accession no. KJ697768, 3123bp), the *CHK1*–942 fragment was used as a probe to isolate *CHK1* from a *P. patens* λZAPII cDNA library (Strepp *et al.*, 1998), according to standard procedures. Full-length *CHK2* (accession no. KJ697769, 3249bp) was achieved by RACE (rapid amplification of cDNA ends) (SMART 5′ and 3′ RACE cDNA amplification kit; Clontech) with gene-specific primers (*cre2* 5′ RACE, gcagtagacggcgaaggtgaaca; and *cre2* 3′ RACE, tgccgtcatagcgaagtctcagt) on Δ*chk1* cDNA. To retrieve *CHK3* (accession no. KJ697770, 3306bp), specific primers (*chk3* for, atgagacaaagaaaacagttgatcaatcc; and *chk3* rev, attcgcctggaagaaatgctttgcaacc) were used to amplify and subclone *CHK3* from cDNA derived from 4-week-old gametophores. Partial sequencing served to prepare a complete cDNA by commercial gene synthesis (GenScript, Piscataway, NJ, USA).

### Cytokinin binding assay

The cytokinin binding assay was performed as has been described previously (Romanov and Lomin, 2009). In brief, the respective cytokinin receptor (*AHK4*, *CHK1*, *CHK2*, and *CHK3*) was cloned into the pDEST15 vector (Invitrogen, Karlsruhe, Germany) and expressed using the *Escherichia coli* strain BL21 (DE) pLys. The empty pDEST15 vector was used as a negative control. Tritium-labeled *trans*-[^3^H]zeatin (*t*Z; 592 GBq mmol^–1^) was obtained from the Isotope Laboratory of the Institute of Experimental Botany (Prague, Czech Republic).

### In planta *complementation assay*

A protoplast transactivation assay (PTA) using protoplasts from the Arabidopsis *ahk2,ahk3* double mutant was conducted as previously described (Choi *et al.*, 2012). In brief, mesophyll protoplasts were isolated from 5- to 6-week-old Arabidopsis plants of the *ahk2,ahk3* double cytokinin receptor knockout. The 350bp promoter fragment of the type-A response regulator *ARR6* was used as a reporter construct and the type-B response regulator *ARR*2 as an effector. As an activator, the cDNAs of *CHK1*, *CHK2*, and *CHK3* as well as that of *AHK4* as a positive control were co-expressed with *ARR2*, respectively. The empty expression vector served as a negative control. The enzyme neuraminidase (NAN) was used as an internal control to standardize expression levels and to calculate relative expression levels (Kirby and Kavanagh, 2002). The details of the PTA protocol and the analysis of the results have been published previously (Ramireddy *et al.*, 2013).

### P. patens *culture*

The sequenced wild-type *P. patens* Hedw. Bruch & Schimp strain used in this study was collected from Gransden Wood, Huntingdonshire, UK in 1968 (Rensing *et al.*, 2008). Standard growth conditions were 25 °C, in white light (100 µE m^−2^ s^−1^) for a light:dark cycle of 16:8h. For transformation and cytokinin profiling, liquid cultures were regularly disintegrated and grown in A′BCD(N)TV medium [0.356mM Ca(NO_3_)_2_, 1.01mM MgSO_4_, 1.84mM KH_2_PO_4_, 10mM KNO_3_, 0.044mM FeSO_4_ supplemented with Hoagland trace element solution (1ml l^–1^) and the vitamins nicotinic acid (8 µM), *p*-aminobenzoic acid (1.8 µM), and thiamine HCl (1.5 µM)] according to Wang *et al.* (1981). For phenotyping, budding assays, and quantitative real-time PCR, cultivation was performed on KNOP agar medium according to Hahn and Bopp (1968).

### Generation of chk knockout mutants

A Δ*chk* mutant collection comprising three single mutants, three double mutants, and one triple mutant was generated by sequential protoplast transformation with gene-disrupting vectors. The targeted loci, details on mutant generation [vector cloning, transformation protocol, and antibiotic selection Supplementary Table S1], as well as characterization [proof of insertion via PCR (see Supplementary Fig. S3) and absence of transcript (via RT–PCR (see Supplementary Fig. S4)], are given as the Supplementary data available at *JXB* online. For the Δ*chk1* mutants, a 300bp fragment has been deleted (scaffold_50:1326711..1326313), for Δ*chk2* 77bp (scaffold_194:351,070..351,147), and for Δ*chk3* 5016bp (scaffold_252:345,051..350,06 (5016bp).

### Budding assay

Budding assays were performed as previously described by von Schwartzenberg *et al.* (2007) after cultivation of protonema for 10 d.

### Cytokinin analysis by UHPLC-MS/MS

Liquid cultures of the wild type and the three double mutants (*Δchk1,2*; *Δchk1,3*; *Δchk2,3*) as well as the triple mutant were grown for 21 d and harvested as previously described (von Schwartzenberg *et al.*, 2007). Three biological replicates were grown separately for the wild type and each mutant line. The extraction and purification was carried out in two technical replicates for each biological replicate. Samples (5mg DW) were homogenized under liquid nitrogen, extracted in modified Bieleski buffer (methanol/ water/formic acid, 15/4/1, v/v/v) (Novák *et al.*, 2008), and then purified using two solid phase extraction columns, a C18 octadecylsilica-based column (500mg of sorbent, Applied Separations) and after that an MCX column (30mg of C18/SCX combined sorbent with cation-exchange properties, Waters) (Dobrev and Kaminek, 2002). Analytes were eluted by two-step elution using a 0.35M NH_4_OH aqueous solution and 0.35M NH_4_OH in 60% (v/v) MeOH solution. Cytokinin levels were determined using ultra high performance liquid chromatography-electrospray tandem mass spectrometry (UHPLC-MS/MS) with stable isotope-labeled internal standards as a reference (Svacinova *et al.*, 2012).

### RNA isolation and real-time PCR

RNA was extracted from the wild type as well as from the three double mutants and the triple mutant using the Trifast Reagent (Peqlab, Germany) according to the manufacturer. After DNaseI (Fermentas, Germany) treatment, cDNA was synthesized using peqGOLD M-MULV H plus (Peqlab). Real-time PCR was performed on a SteponePlus cycler (Applied Biosystems) using gene-specific primers and KAPA SYBR FAST Universal (Peqlab). Ribosomal protein L21 (Wang and Irving, 2011) was used as an endogenous control, and a primer efficiencies >95% were established for all targets (primers are given in Supplementary Table S2 at *JXB* online). Calculations were performed using the Stepone Software V. 2.3 with the ΔΔCt method.

## Results

### *CHK1 and CHK2 bind* trans*-zeatin in an* in vivo *binding assay*

**Fig. 1.**
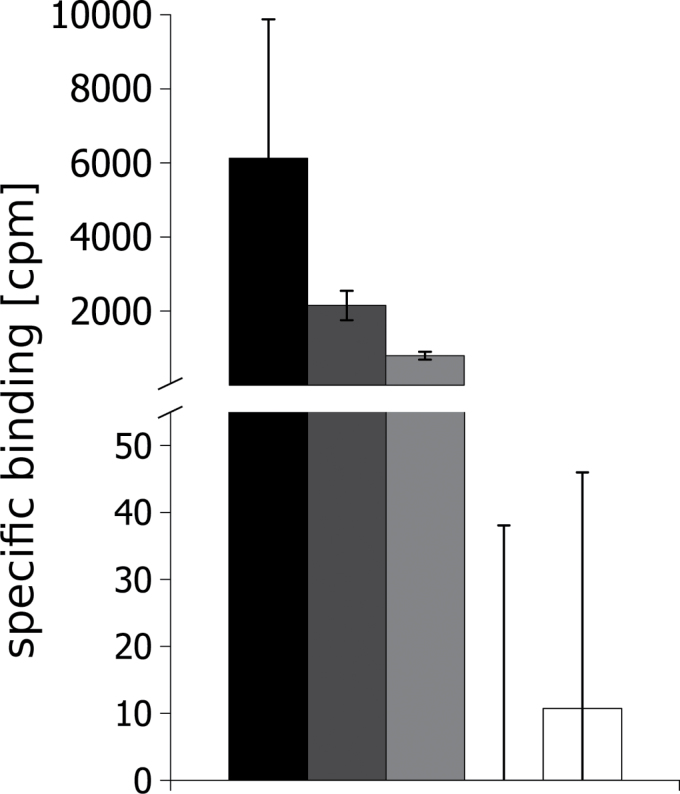
CHK1 and CHK2 bind *t*Z in an *in vivo* cytokinin binding assay. All receptors were expressed as GST fusion proteins in *E. coli* strain BL21 (DE) pLys. The specific binding to *trans*-[2-^3^H]zeatin was analyzed according to Romanov and Lomin (2009). Shown are biological replicates (*n*=3) and their SD (error bars). Expression of the different fusion proteins was confirmed by western blot (see Supplementary Fig. S1 at *JXB* online).

The sequences for the three *CHK*-coding sequences were retrieved by PCR cloning and submitted to the NCBI (*CHK1*, KJ697768; *CHK2*, KJ697769; and *CHK3*, KJ697770). In order to test the functionality of the three cytokinin receptors, we employed a cytokinin binding assay (Suzuki *et al.*, 2001; Yamada *et al.*, 2001; Romanov and Lomin, 2009). The cloned receptors and the respective controls were expressed in *E. coli* and the binding of radiolabeled *t*Z was tested. The assay was performed with AHK4 and the empty vector as positive and negative controls, respectively (Romanov *et al.*, 2005; Heyl *et al.*, 2007). CHK1 and CHK2 showed binding of *t*Z that was clearly above background (Fig. 1). Surprisingly, in this assay we did not detect any *t*Z binding for CHK3 although the protein was expressed in sufficient quantities (see Supplementary Fig. S1 at *JXB* online).

### *CHK1 and CHK2 function as cytokinin receptors in an* in planta *complementation assay*

**Fig. 2.**
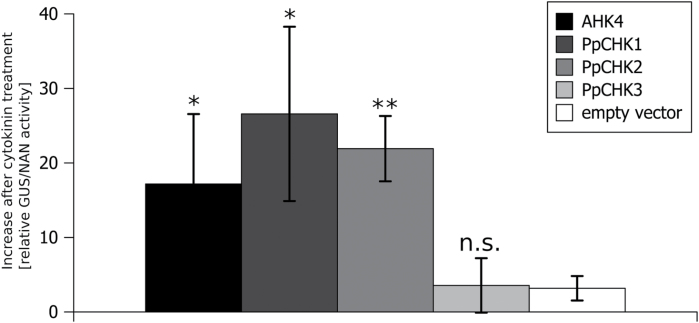
*P. patens* cytokinin receptors PpCHK1 and PpCHK2 activate the cytokinin-dependent TCS in *ahk2-5,ahk3-7* double knockout mutant of Arabidopsis. Cytokinin perception-deficient protoplasts (*ahk2-5,ahk3-7*) (Riefler *et al.*, 2006) were co-transformed with the cytokinin-responsive *ARR2* (effector), the *ARR6* promoter fused to β-glucuronidase (reporter), 35S::NAN (internal reference), and the indicated cytokinin receptor (activator) under the control of the 35S promoter. Protoplasts were incubated overnight with and without *trans*-zeatin; subsequently *ARR6* promoter *trans*-activation was measured. Results were normalized by the internal reference, and the specific activity upon cytokinin treatment was calculated (normalized reporter activity with cytokinin minus normalized reporter activity without cytokinin). Depicted results are mean values of three biological replicates, and whiskers represent the SD (*n*=3, mean ±SD, *t*-test different from vector control, *P<0.05; **P<0.005; n.s., not significant).

In order to test the *in vivo* functionality of the three cytokinin receptors, we employed an *in planta* complementation assay in which a candidate receptor is expressed in protoplasts from Arabidopsis plants in which two of the three cytokinin receptors are mutated (*ahk2,ahk3*) (Choi *et al.*, 2012). The complementation was quantified using a β-glucuronidase (GUS) reporter gene. All genes were individually expressed under the control of the 35S promoter and the cells were treated with *t*Z. Using the cytokinin receptor AHK4 from Arabidopsis as a positive control, *ahk2,ahk3* was complemented as described previously, while the empty vector as negative control showed only a weak activation of the reporter gene (Fig. 2). Of the three cytokinin receptors, only CHK1 and CHK2 showed a complementation in the double mutant. In fact, they complemented the *ahk2,ahk3* double mutant even better than AHK4. However, no receptor activity was detected in this assay for CHK3 as compared with the controls (Fig. 2).

Taking the results of the binding and complementation assay together, it was shown that at least two of the three classical CHK proteins fulfill the requirements to function as a cytokinin receptor.

### *Generation of Δ*chk *knockout mutants*

In order to characterize the *in planta* function of the CHK1, CHK2, and CHK3 receptors in *P. patens*, a mutant collection comprising single (Δ*chk1*, Δ*chk2*, and Δ*chk3*), double (Δ*chk1,2*; Δ*chk1,3*; and Δ*chk2,3*), and triple (Δ*chk1,2,3*) mutants was generated by protoplast transformation using gene targeting constructs for each locus. Detailed information of the generation and characterization of this collection are provided in Supplementary Fig. S2 at *JXB* online. Each of the constructs harbored a different resistance cassette (for selection on G418, hygromycin B, and zeocin, respectively), thus enabling selection of plants with up to three *CHK* loci targeted. Mutants were analyzed by detailed PCR-based characterization of genomic DNA (see Supplementary Fig. S3) and cDNA (see Supplementary Fig. S4) which proved that (i) the respective *CHK* loci were targeted and (ii) the corresponding transcripts were no longer detectable. Furthermore, it was confirmed that the mutants had maintained the haploid status using flow cytometry (not shown).

### *Phenotype of* CHK *knockout mutants: protonema and gametophore development*

**Fig. 3.**
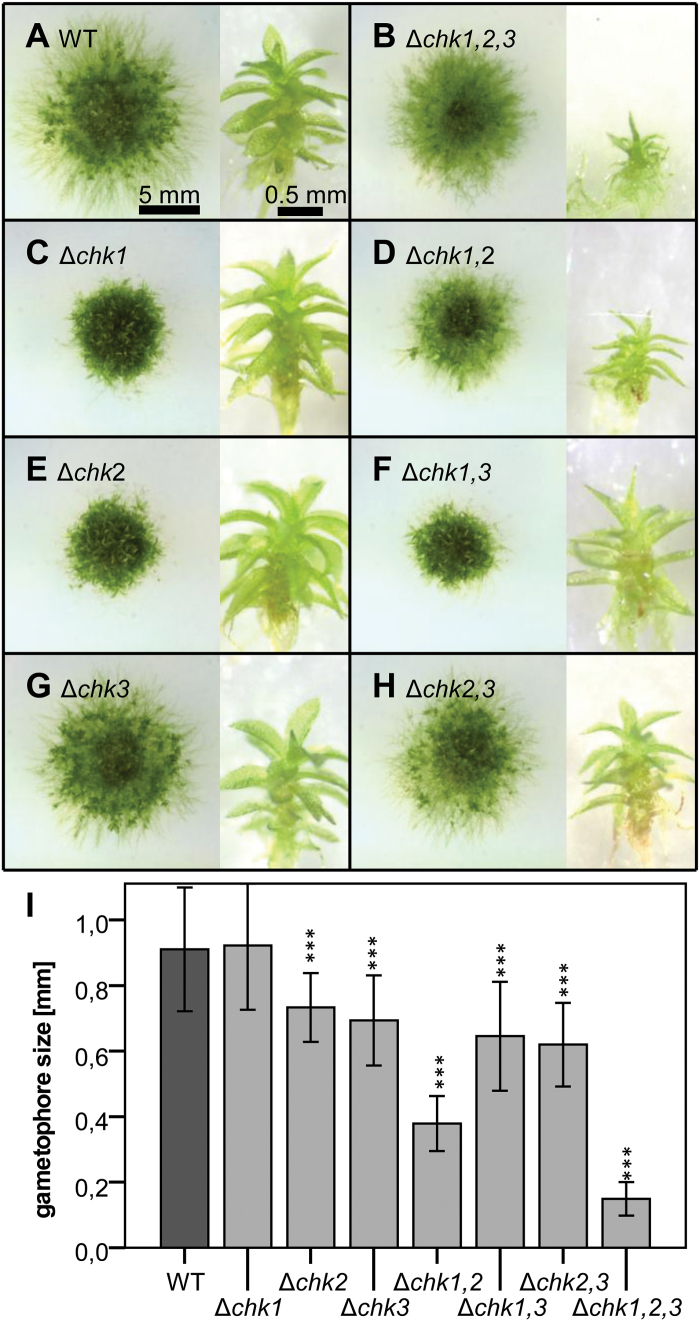
(A–H) Phenotypes of single, double, and triple mutants of *CHK1*, *CHK2*, and *CHK3*. Each panel shows moss colonies (7 weeks old) on the left and isolated gametophores (14 weeks old) on the right. Cultures were grown on KNOP agar medium. Corresponding pictures were taken at the same magnification; scale bars are given in (A). (I) Mean size of gametophores after 14 weeks [mean ±SD, *t*-test different from the wild type (WT), ***P<0.001].

Knockout of a single receptor in Δ*chk1* or Δ*chk2* altered the growth morphology of moss grown on agar medium. Wild-type colonies showed a large area with undifferentiated protonema in the outer parts, and displayed bud and gametophore formation in the inner parts. The Δ*chk1* and Δ*chk2* single mutants had a smaller colony diameter and fewer protruding protonema. In contrast, Δ*chk3* did not exhibit a reduction of the colony size (Fig. 3C, E, G). For the double mutants, the colony size was most strongly reduced for Δ*chk1,3* (Fig. 3D, F, H). Detailed data on colony diameter over 6 weeks are given in Supplementary Fig. S5A at *JXB* online).

While the gametophores formed by Δ*chk1* mutants had an average size comparable with the wild type, the size of gametophores of Δ*chk2* and Δ*chk3* was reduced. The size of the gametophores formed by Δ*chk1,2* was drastically reduced compared with the wild type and the single mutants (Fig. 3D). Gametophores of the Δ*chk1,3* and the Δ*chk2,3* mutants were smaller compared with the wild type, although they were larger than gametophores of Δ*chk1,2*. This indicates the relevance of CHK1 and CHK2 for gametophore development. The development of gametophores in the double mutants occurred as for the wild type within 2 weeks of culture (Supplementary Fig. S5B at *JXB* online). All three *CHK* double mutants eventually developed antheridia and archegonia, produced a sporophyte after water-mediated fertilization, and finally completed the entire life cycle with the germination of haploid spores.

The triple receptor mutant Δ*chk1,2,3* showed a minor reduction in colony diameter compared with the wild type. However, the number of gametophores per colony was reduced as the colony consisted mainly of protonema. Gametophore formation was delayed by ~1 week (Supplementary Fig. S5B at *JXB* online). Furthermore, even after 14 weeks, the size of the gametophores was <20% of the size of the wild type, indicating the importance of all three receptors not only for bud initiation but also for gametophore development (Fig. 3B). This result functionally links two essential processes in the development of the moss, namely the onset of budding and gametophore formation, to the cytokinin receptors. Moreover, no antheridia and archegonia were observed for the triple mutants which arrested their life cycle at the gametophore stage, further highlighting the importance of the classical cytokinin receptors for the life cycle of *P. patens*.

### CHK *mutants display an altered cytokinin tolerance and response*

**Fig. 4.**
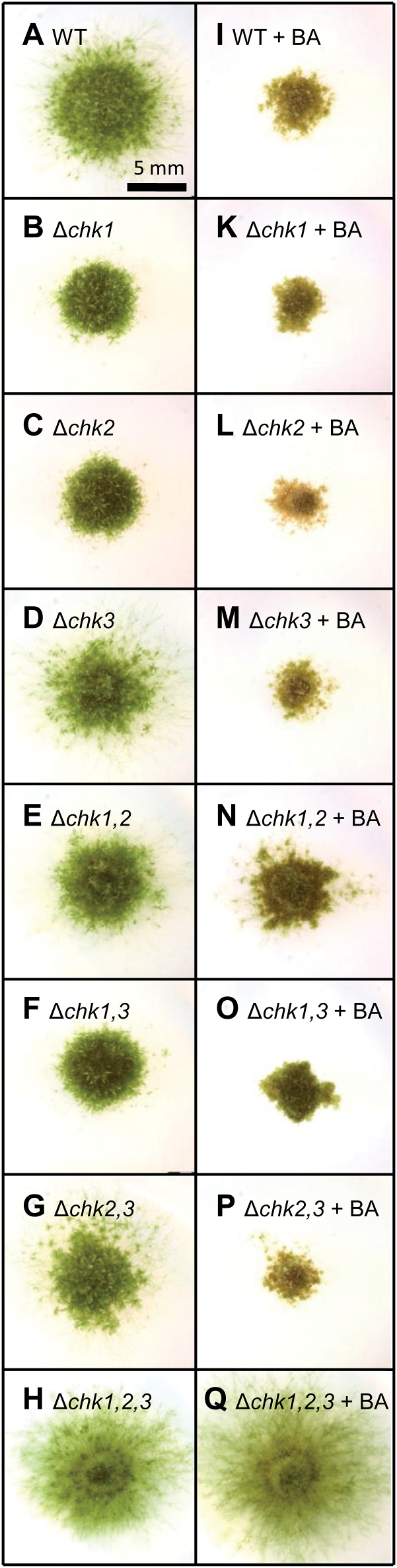
Tolerance of single, double, and triple mutants of *CHK1*, *CHK2*, and *CHK3* to a high dose of cytokinin in comparison with the wild type. Seven-week-old cultures grown on KNOP agar plates. (A–H) No exogenous cytokinin, (I–Q) 1 µM benzyladenine (BA). All pictures are at the same scale; the scale bar is given in (A).

Next, we investigated the effect of cytokinin treatment on the different mutant lines in a cytokinin tolerance assay. Plants of the *CHK* mutant collection were inoculated on KNOP agar medium supplemented with 1 µM benzyladenine (BA), representing a concentration far beyond the range measured for endogenously produced cytokinins in *P. patens* (von Schwartzenberg *et al.*, 2007). We have further chosen BA for this experiment as it is less prone to degradation by cytokinin oxidase/dehydrogenase compared with isoprenoid cytokinins (Avalbaev *et al.*, 2012). At a concentration of 1 µM BA, the protonemal growth of the wild type was strongly inhibited, and malformed buds developed. In contrast, the high dose of BA did not lead to growth reduction and bud formation in the Δ*chk1,2,3* triple mutant, indicating a strong cytokinin insensitivity of this mutant (Fig. 4).

The exposure of the other members of the *chk* mutant collection to high doses of BA showed that the presence of a single CHK receptor is sufficient to confer sensitivity to an excess of cytokinin (Fig. 4). While all single and double mutant genotypes showed a brown or pale color, the protonema of the triple Δ*chk1,2,3* mutant was not visibly affected in pigmentation by the BA overdose. The tolerance assay showed that all three classical CHK receptors are involved in growth inhibition as well as in the formation of malformed buds, which are typical responses of *P. patens* to a high dose of cytokinin (von Schwartzenberg, 2009).

### *Budding bioassay reveals differences in the biological roles of* CHK1, CHK2, *and* CHK3

**Fig. 5.**
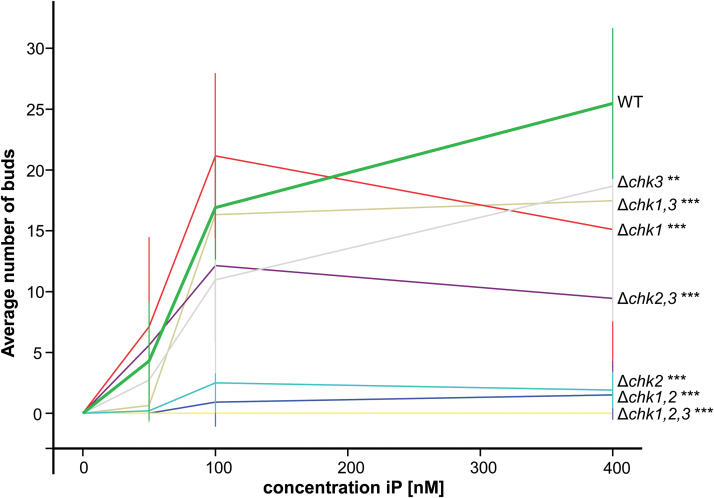
Dose-dependent budding response to isopentenyladenine (iP) in the wild type (WT), and single, double, and triple Δ*chk* mutants. Equal amounts of protonema were suspended on KNOP agar medium containing 0–400nM iP, and bud formation was analyzed microscopically after 10 d under standard conditions. The average number of buds corresponds to one microscopic view field (3.8mm^2^). At least two different biological replicates were counted in 5–10 view fields (mean ±SD, *t*-test different from the WT at 400nM, **P<0.01, ***P<0.001; the complete data set is given in Supplementary Table S3 at *JXB* online.

Cytokinins affect many aspects in the development of mosses (for a review, see von Schwartzenberg, 2009), with the induction of buds being the most striking. In order to establish whether one or more of the three CHKs under investigation are involved in this process, the *CHK* mutant collection was tested in a dose-dependent budding assay (Hahn and Bopp, 1968). The number of buds was counted after 10 d of growth on different concentrations of isopentenyladenine (iP; 50, 100, and 400nM). For the genotypes Δ*chk1*, Δ*chk3*, Δ*chk1,3*, and Δ*chk2,3*, a slightly reduced budding was observed (Fig. 5); however, the high variability of these bioassays results should be taken into account. For the genotypes Δ*chk2* and Δ*chk1,2* only minimal bud formation was recorded. These genotypes only responded to concentrations of iP >50nM (Supplementary Table S3 at *JXB* online), whereas in the wild type and most of the other mutants bud induction was already clearly detectable at 50nM iP. Strikingly, in this bioassay, the triple mutant Δ*chk1,2,3* did not exhibit any budding response.

From the results for the different double mutant combinations tested in the budding assay it can be deduced that CHK1 and CHK2 alone are capable of mediating budding as a response to increased iP concentrations. The response to iP in the presence of CHK1 alone (in Δ*chk2,3*) was slightly weaker than in the presence of CHK2 alone (in Δ*chk1,3*). Only a very low budding response was observable in the double mutant Δ*chk1,2* mediated by CHK3 alone. Noticeably, the CHK3 receptor in the absence of CHK2 and CHK1 was insufficient to transduce the iP signal in order to result in significant budding (Fig. 5). In summary all three receptors participate in the budding response in this short-term assay. The absence of all three receptors leads to a complete lack of cytokinin-dependent bud induction, thus indicating an essential role for the CHKs in this developmental transition.

### Differential budding in response to distinct cytokinins

**Fig. 6.**
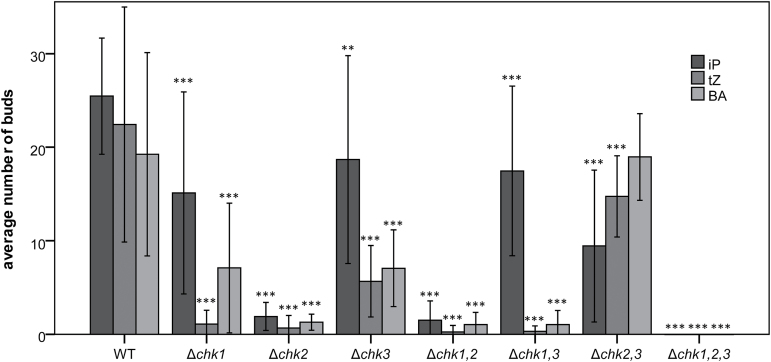
Budding response of the wild type (WT), and double and triple Δ*chk* mutants to iP, *t*Z, and BA. The budding response of the different genotypes was assessed after 10 d on KNOP agar medium supplemented with 400nM iP, *t*Z, and BA, respectively. Equal amounts of protonema were suspended on KNOP agar medium and bud formation was analyzed microscopically after 10 d under standard conditions. The number of buds corresponds to one microscopic view field (3.8mm^2^). At least two different biological replicates were counted in 5–10 view fields (mean ±SD, *t*-test different from the WT for each individual cytokinin, **P< 0.01, ***P<0,001); the complete data set is given in Supplementary Table S3 at *JXB* online).

In order to investigate how the three CHK receptors differ in their response to different cytokinins, we performed the budding assay with the three double mutants as well as the triple mutant using the cytokinins iP, *t*Z, and BA (each at 400nM, Fig. 6) known to be the most active in this assay (von Schwartzenberg *et al.*, 2007). As determined in the Δ*chk2,3* mutant background, the receptor CHK1 alone is capable of mediating a budding response to all applied cytokinins. In Δ*chk1,3*, where only the CHK2 receptor is present, the budding response was high with iP but strongly impaired for *t*Z and BA—indicating a preference for iP. A strongly reduced response for all the three cytokinin bases was noted for the Δ*chk1,2* double mutant, indicating that CHK3 alone is not very active—at least in the protonemal stage. No budding response at all was found for the Δ*chk1,2,3* triple mutant no matter which of the three cytokinins was applied (Fig. 6).

### *Relative expression of *CHK* genes in Δ*chk *mutant backgrounds*

**Fig. 7.**
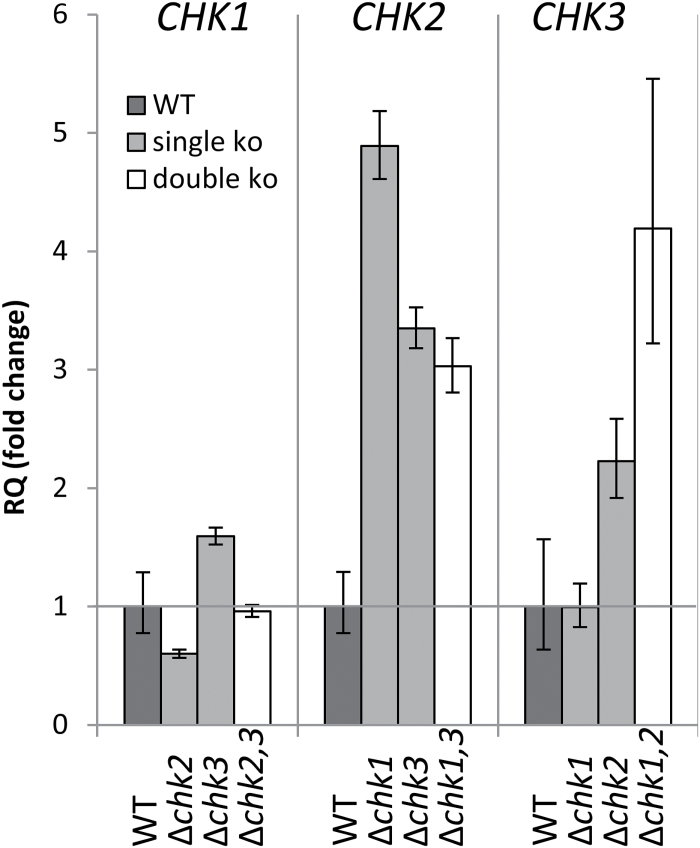
Relative expression of *CHK1*, *CHK2*, and chk3 in the respective single and double mutants. Three biological replicates were measured (7-day-old plants grown on KNOP agar, under standard conditions) with three technical replicates. 60S ribosomal protein L21 (Wang and Irving, 2011) served as the endogenous control, and gene expression was normalized to wild-type (WT) levels. Analysis of RQ (relative quantities) was performed with StepOne Plus software (Life Technonolgies^®^). Mean ±SD from technical replicates. *t*-test for differences from the WT for each target among biological replicates yielded no significant differences.

In order to investigate compensatory interaction between the different receptors on the transcript level, quantitative real-time-based analysis of *CHK* gene expression in the mutants and wild type was carried out. This analysis revealed that *CHK1* expression is not or is only slightly affected by the knockout of *CHK2*, *CHK3*, or both receptors. However, expression of *CHK2* seems to be 3- to 5-fold up-regulated in the single, and the double mutants of *CHK1* and *CHK3* compared with the expression level measured for the wild type. The expression of *CHK3* was found to be up-regulated in Δ*chk2* and Δ*chk1,2,* but not in Δ*chk1* (Fig. 7). Thus while the expression of *CHK1* is quite stable regardless of the genetic background, the transcript level of both *CHK2* and *CHK3* increased in most receptor mutant backgrounds.

### *Cytokinin profiles of Δ*chk *mutants*

**Fig. 8.**
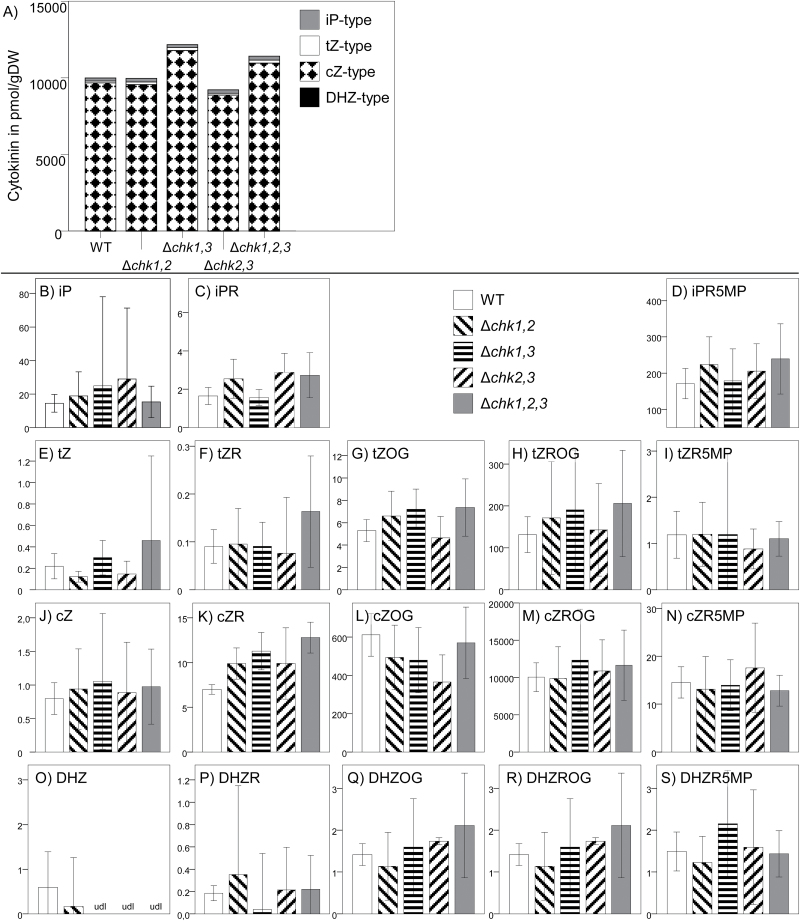
Average level of isoprene-type cytokinins in tissue of *P. patens* wild type and the cytokinin receptor mutants Δ*chk1,2,3* (triple) and Δ*chk1,2*; Δ*chk1,3* and Δ*chk2,3* (double). Each genotype was cultured three times independently for 21 d, sampled at three independent time points, and measured by UHPLC-MS/MS (*n*=9–24). Data present mean values with 95% confidence intervals. Cytokinin content in the mutants was compared with that in the wild type by independent samples Kruskal–Wallis test (confidence interval 95%, significance level 0.5); no significant changes compared in all samples with the wild type. Values are given in pmol g^–1^ DW. (A) Sum of all measured cytokinins. (B–S) Levels of each individual cytokinin; columns are the same as indicated in (A). *t*Z, *trans*-zeatin; *t*ZOG, *trans*-zeatin *O*-glucoside; *t*ZR, *trans*-zeatin riboside; *t*ZRMP, *trans*-zeatin riboside-5′-monophosphate; *t*ZROG, *trans*-zeatin riboside *O*-glucoside cZ, *cis*-zeatin; *c*ZOG, *cis*-zeatin *O*-glucoside; *c*ZR, *cis*-zeatin riboside; *c*ZRMP, *cis*-zeatin riboside-5′-monophosphate; *c*ZROG, *cis*-zeatin riboside *O*-glucoside; DHZ, dihydrozeatin; DHZOG, dihydrozeatin *O*-glucoside; DHZR, dihydrozeatin riboside; DHZRMP, dihydrozeatin riboside-5′-monophosphate; DHZROG, dihydrozeatin riboside *O*-glucoside; iP, *N* ^6^-isopentenyladenine; iPR, *N* ^6^-isopentenyladenosine; iPRMP, *N* ^6^-isopentenyladenosine-5′-monophosphate. udl, under the detection limit.

The transcriptional response of *CHK2* and *CHK3* to a receptor deficiency led to the question of whether there is a connection between cytokinin signaling and metabolism in *P. patens*. Previously, it has been shown in Arabidopsis that deficiencies in cytokinin receptors can result in changes of cytokinin homeostasis (Riefler *et al.*, 2006). Thus we established the cytokinin profiles of the three double mutants (Δ*chk1,2,* Δ*chk1,3*, and Δ*chk2,3*) and the triple mutant Δ*chk1,2,3* using UHPLC-MS/MS measurements and compared them with the profile of the wild type (Fig. 8). Each of the genotypes was cultured three times independently as a protonemal culture in liquid medium, and two technical replicates were made for each extract. Although there were individual changes, a general increase of all types of cytokinins, as described for Arabidopsis (Riefler *et al.*, 2006), was not found for the moss mutants. *cis*-Zeatin riboside *O*-glucoside (*c*ZROG), which is by far the most abundant cytokinin in *P. patens* (von Schwartzenberg *et al.*, 2007), was only found at slightly higher levels in the Δ*chk1,3* mutant. However no significant changes in cytokinin levels were measured in the cytokinin receptor mutants when compared with the wild type.

## Discussion

The aim of this study was to characterize the properties and biological roles of the three classical cytokinin receptors of *P. patens*. In order to test the functionality of the three cloned receptors *CHK1*, *CHK2*, and *CHK3*, we used two well-established cytokinin receptor assays, the cytokinin binding assay and the *in planta* complementation assay (Mizuno and Yamashino, 2010; Choi *et al.*, 2012). The assays confirmed the activity of CHK1 and CHK2 in hormone binding (Fig. 1), as well as their translation into downstream signaling, at least in Arabidopsis (Fig. 2). To our surprise, for CHK3 no activity was confirmed in either of these assays (Fig. 1, 2). However, this does not mean that the third receptor is not functional in *P. patens*. In fact the analysis of the knockout lines clearly shows that CHK3 has a role as a cytokinin receptor in the moss.

### *CHK receptors are functional* in planta

To confirm the function of CHKs as cytokinin receptors in *P. patens*, different cytokinin-dependent assays using a collection of receptor knockout mutants were conducted. In flowering plants it is known that high concentrations of cytokinins can induce senescence and programmed cell death (Carimi *et al.*, 2003; Vescovi *et al.*, 2012). Our experiments confirmed the growth-inhibiting effect of 1 µM BA in *P. patens* (Thelander *et al.*, 2005). We clearly demonstrated that this cytokinin-dependent growth inhibition is mediated via the CHK receptors, as the Δ*chk1,2,3* triple mutant was not affected by a high dose of BA. The cytokinin tolerance assay (Fig. 4) confirmed a role for CHK3 in cytokinin perception as the Δ*chk1,2* double mutant, with only CHK3 left as a functional receptor, was more affected by BA than plants with a simultaneous knockout of all three classical receptors (Δ*chk1,2,3*). We deduce from the *in planta* experiments that CHK3 is capable of reacting to BA. The absence of a *t*Z-mediated response in the complementation and binding assays could be explained by either an incorrect protein processing in a heterologous system or a general low functionality of CHK3. While a *t*Z binding and a *t*Z-dependent response of CHK3 remains unclear, it can be stated that CHK3 mediates a clear iP response in the budding assay (Fig. 6) and a clear BA response in the tolerance assay (Fig. 4).

This experiment also indicates that apart from CHK1, CHK2, and CHK3, no other receptor is necessary to confer sensitivity to a cytokinin overdose, at least at this growth stage of the moss (Fig. 4).

A more detailed analysis using the well characterized cytokinin-dependent budding response (Hahn and Bopp, 1968) revealed that each CHK receptor, including CHK3, mediates a cytokinin response *in planta* as all three double mutants respond to cytokinins. CHK2 has a prominent role in this developmental process as all mutants in which this cytokinin receptor was missing showed a much weaker cytokinin response and (almost) no response to low levels of iP (50nM; Fig. 5; Supplementary Table S3 at *JXB* online) when compared with those missing CHK1 or CHK3. In contrast, the mutants Δ*chk1*, Δ*chk3*, Δ*chk1,3*, and Δ*chk2,3* were less affected in budding frequency, also at high cytokinin concentrations (Fig. 5). In order to check if these effects depend on the type of cytokinin used, the assay was extended using three different cytokinins at 400nM (Fig. 6). These experiments showed weak budding when only CHK3 was present regardless of the cytokinin used, which indicates that this receptor is functional, but does not play a critical role for the transition from protonema to gametophore during the moss life cycle. In contrast, CHK1 responded to a broader cytokinin spectrum as all three tested cytokinins led to bud formation. Only CHK2 showed a preference for one particular cytokinin, iP, as this cytokinin had a far stronger bud-inducing effect than *t*Z or BA in the Δ*chk1,3* mutant (Fig. 6). We conclude from the *in planta* cytokinin response assays that all three receptors mediate a cytokinin-dependent signal independently from each other. Despite its responsiveness towards high doses of cytokinin, CHK3 seems to be of only minor importance for bud formation. CHK1 and CHK2 play a major role in triggering this developmental process, however with differences in the cytokinin preference.

The differences and redundancies among the investigated cytokinin receptors were further highlighted by the changes in their expression in the different mutant backgrounds. The peculiar finding of a higher sensitivity of the Δ*chk2,3* mutant compared with the Δ*chk2* mutant to iP can be explained by taking into account the expression level of *CHK1*. In Δ*chk2*, but not in the Δ*chk2,3* mutant, the *CHK1* level is reduced, which could contribute to the reduced sensitivity of Δ*chk2*. However, in what way and to what extent the regulation and redundancies among the receptors are realized remain to be investigated. The *CHK2* and *CHK3* transcript levels were elevated in mutants still expressing the respective receptor. Thus it seems possible that the relatively small loss of bud formation observed in Δ*chk1,3* (Fig. 5) was partly due to an up-regulated *CHK2* expression in this mutant. The expression levels of *CHK1* either remained constant or increased slightly in Δ*chk2* mutants (Fig. 7).

### P. patens *development depends on functional CHK receptors*

The phenotypic characterization of colony shape and gametophore size revealed differences between the mutants and wild type. Single mutants expressed minor phenotypic changes (reduced colony diameter of Δ*chk1* and Δ*chk2*) or were even indistinguishable from the wild type (Δ*chk3*), thus indicating again that CHK3 is not a major player for cytokinin perception in *P. patens*. The apparent minor role of CHK3 was further corroborated by the similar phenotype of the Δ*chk1,2* double mutant and the triple mutant Δ*chk1,2,3*, which both exhibited reduced differentiation and a reduction of gametophore size. However, the CHK3 receptor possesses enough activity to provide basal functions in cytokinin activity as the Δ*chk1,2* double mutant like the Δ*chk1,3* and Δ*chk2,3* mutants, is able to undergo its entire life cycle including the formation of gametangia, sporophyte, and viable spores (not shown).

The importance of the cytokinin receptors for sexual reproduction was further emphasized by the inability of the triple mutant to form sporophytes. A similar phenotype was also observed when the cytokinin oxidase gene *AtCKX2* was overexpressed in *P. patens*. These plants with a lowered content of cytokinins also showed a reduced budding response and absence of sporophyte formation (von Schwartzenberg *et al.*, 2007). The life cycle of *P. patens* is apparently dependent on a functionality in the level of cytokinin homeostasis as well as of cytokinin perception.

A detailed developmental analysis of vegetative and generative stages in CHK mutants employing approaches such as presented by Coudert *et al.* (2015) and Landberg *et al.* (2013) is the subject of ongoing studies.

### Classical CHKs play a key role in cytokinin perception and moss development

The experiments of this study clearly demonstrate the crucial role of CHK1, CHK2, and CHK3 for cytokinin perception and especially for the cytokinin-triggered formation of buds in moss. Recently, an additional subfamily of cytokinin receptors has been discovered (Gruhn *et al.*, 2014). While sharing the same overall domain structure with the classical CHKs, their CHASE domain shows a lower conservation compared with the classical cytokinin receptors (Gruhn *et al.*, 2014). One of the eight members of the new *P. patens* CHK subfamily, CHK4, has been characterized, and cytokinin binding and cytokinin-dependent activation of a two-component signaling chain was shown. The results of the analysis presented in this study of the classical Δ*chk* mutants raise the question of the biological role of the receptors of the new subfamily. In particular, the facts that the triple mutant was completely resistant to the applied cytokinins in the tolerance assay and that bud formation was strongly delayed seem to indicate that these new receptors might not be critical in the cytokinin biology of the moss. However, one has to consider that budding is not the only developmental process regulated by cytokinin in *P. patens* and that small gametophores did form eventually in the Δ*chk1,2,3* triple mutant. Furthermore the transition from chloronema to caulonema, the formation of secondary chloronema, and the development of brachycytes and tnema cells, amongst other processes, are influenced by cytokinin (von Schwartzenberg, 2009). It will be interesting to test if those processes are also affected in the Δ*chk1,2,3* mutant or if the newly identified receptors function in one or more of these developmental processes. While the biological function of these ‘novel’ CHKs in not yet clear, their evolutionary origin is clearly different from those of the other cytokinin receptors (Gruhn *et al.*, 2014). Interestingly, sequences from charophyceae algae [i.e. EST (expressed sequence tag) evidence from *Spirogyra pratensis* and the sequenced genome of *Klebsormidium flaccidum*] clustered between both clades of *CHK*s and those might be ancestral to both (Gruhn *et al.*, 2014; Hori *et al.*, 2014; E. Kaltenegger and A. Heyl, unpublished data). It is conceivable that they represent an ancestral type of cytokinin receptor that evolved into the classical CHK receptors found in land plants (e.g. PpCHK1, AtAHK4, and others).

### *Comparison of cytokinin signaling between* P. patens *and Arabidopsis*

Given the importance of cytokinin as a regulator of plant growth and development (Hwang *et al.*, 2012; Kieber and Schaller, 2014), it is surprising that *A. thaliana* is the only species in which cytokinin receptors have been analyzed systematically.

On the protein level, the classical CHK receptors from Arabidopsis and *P. patens* show a high degree of similarity with respect to their domain architecture of the whole protein and on the sequence level, also within the CHASE domain (Gruhn *et al*., 2014, 2015). These structural similarities also translate to functional similarities as we were able to complement *ahk2,ahk3* deficiency in Arabidopsis protoplasts by transient expression of *PpCHK1* and *PpCHK2* (Fig. 1).

This study investigated the classical cytokinin receptors of the moss *P. patens* in detail, thus allowing phenotypic comparisons of cytokinin receptor mutants of an early diverging and a flowering plant. In both cases, the single mutants showed weak or no phenotypes, indicating a high level of redundancy among the receptors. While in Arabidopsis the simultaneous knockout of all cytokinin receptors (*ahk2,ahk3,ahk4*) leads to a severe dwarf plant (Higuchi *et al.*, 2004; Nishimura *et al.*, 2004; Riefler *et al.*, 2006), the respective *P. patens* Δ*chk1,2,3* mutant showed a protonemal growth area that is comparable with that of the wild type. However, gametophores which appeared delayed in the Δ*chk1,2,3* triple mutant when compared with the wild type also exhibited a strong dwarf phenotype (Fig. 3, Supplementary Fig. S5B at *JXB* online). Both the Arabidopsis triple mutant shoot and the *P. patens* triple mutant gametophore are strongly impaired in growth and development. Further, both plants are highly resistant to exogenous cytokinin treatment. In both plant species, different mutant combinations show a distinct response to certain cytokinins, indicating different biological roles for the respective receptors (Higuchi *et al.*, 2004; Nishimura *et al.*, 2004; Riefler *et al.*, 2006; Stolz *et al.*, 2011). Despite the similarities in the cytokinin signaling mechanisms of flowering plants and bryophytes, we uncovered differences in the impacts of cytokinin signaling on the homeostasis of these hormones. In Arabidopsis, it has been shown that deficiency in cytokinin perception results in drastic changes of cytokinin homeostasis; notably, with increasing number of deleted receptors, a significantly increased concentration of numerous cytokinin species was found. In the Arabidopsis triple receptor mutant, there was, for example, a 15-fold increase of *t*Z compared with the wild type (Riefler *et al.*, 2006). The levels of active cytokinins in *P. patens* [iP, iPR, *t*Z, *t*ZR, and dihydrozeatin (DHZ) (von Schwartzenberg *et al.*, 2007)] were not significantly altered in the analyzed Δ*chk* mutants (Fig. 8). Thus the relatively small reduction in budding response for Δ*chk1*, Δ*chk3*, or Δ*chk1,3*, for example (Fig. 5), cannot be explained by an increased production of active cytokinins to compensate the loss of function in the receptor system. In summary, in the moss *P. patens*—in contrast to flowering plants—there is only a minor contribution of the CHK1, CHK2, and CHK3 receptors to cytokinin homeostasis.

### Conclusions

The study presented reveals that at the evolutionary stage of bryophytes, cytokinin signaling is fully established and uses classical receptors of the CHK gene family. The different experiments highlight the common and different properties of the receptors and their roles in developmental processes such as bud formation and gametophore development. The results of this study demonstrate the functionality of the classical PpCHK receptors, which are crucial for key steps in the life cycle of *P. patens.* Currently studies are under way to investigate the impact of the CHK receptors on multiple physiological and developmental aspects, and we expect that this and the presented research will contribute to the understanding of how hormonal regulation was established at the level of bryophytes.

## Supplementary data

Supplementary data are available at *JXB* online.

Supplementary Fig. S1. Expression control of the fusion proteins analyzed in Fig. 1.

Supplementary Fig. S2. Overview of the *chk* knockout plants generated by transformation with the listed vectors.

Supplementary Fig. S3. PCR screening of knockout mutants.

Supplementary Fig. S4. RT–PCR screen of the different mutant lines.

Supplementary Fig. S5. (A) Average colony radius; (B) time course of gametophore frequency.

Supplementary Table S1. Generation of the *chk* mutant collection.

Supplementary Table S2. Primer sequences.Supplementary Table S3. Data for the budding assay (Figs 5, 6).

Supplementary Data
