# Supplementary material for: CHASE domain-containing receptors play an essential role in the cytokinin response of the moss Physcomitrella patens
Source: J Exp Bot. 2015 Nov 23;67(3):667–79. doi: 10.1093/jxb/erv479 (PMC4737067; doi:10.1093/jxb/erv479)
Supplement: Supplementary Data [file supp_67_3_667__index.html]

CHASE domain-containing receptors play an essential role in the cytokinin response of the moss Physcomitrella patens — Supplementary Data 

# CHASE domain-containing receptors play an essential role in the cytokinin response of the moss *Physcomitrella patens*

## Supplementary Data

Data files

- supplementary\_figures\_S1\_S5\_\_\_Tables\_S1\_S3.pdf - Supplementary Data
