# Supplementary material for: CHASE domain-containing receptors play an essential role in the cytokinin response of the moss Physcomitrella patens
Source: J Exp Bot. 2015 Nov 23;67(3):667–79. doi: 10.1093/jxb/erv479 (PMC4737067; doi:10.1093/jxb/erv479)
Supplement: Supplementary Data [file supp_erv479_supplementary_figures_S1_S5___Tables_S1_S3.pdf]

Supplementary data

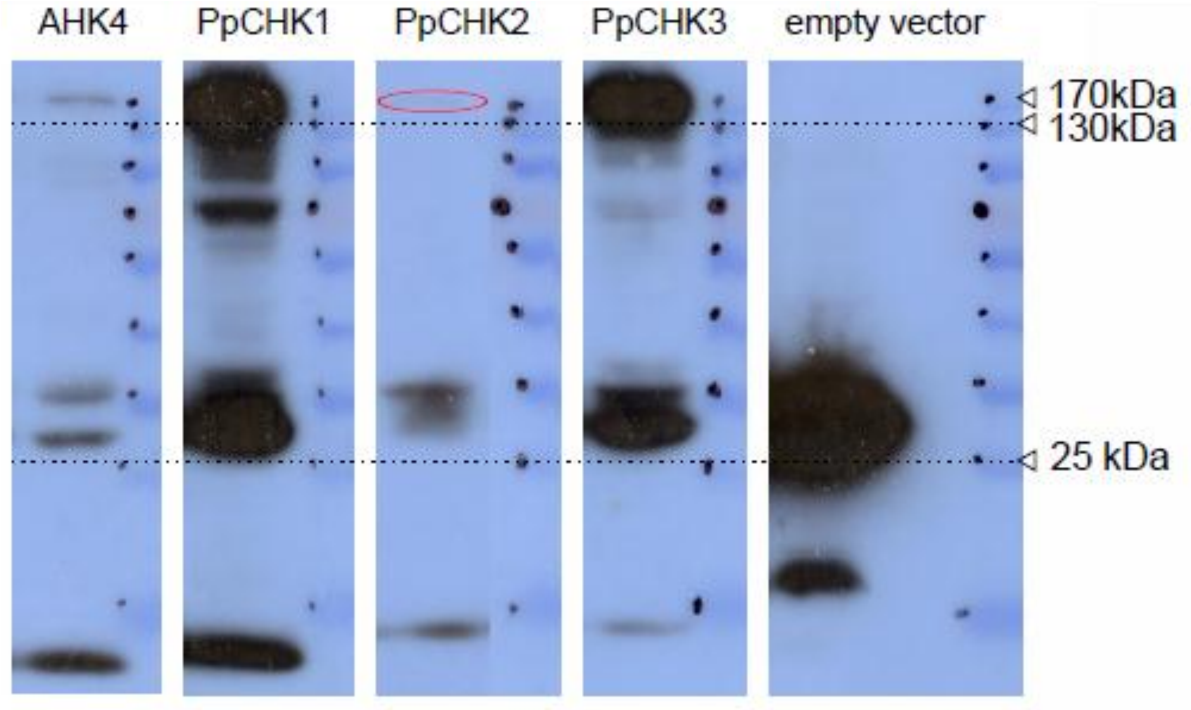

Fig. S 1. Expression control of the fusion proteins analysed in Fig. 1. Immunoblot detection was carried out using the pellet of 1 mL bacterial culture that was separated by SDS-PAGE, blotted to a PVDF membrane and GST-containing bands were stained using a GST specific antibody. White arrowheads mark the position of the protein marker. Encircled in red is the band for PpCHK2.

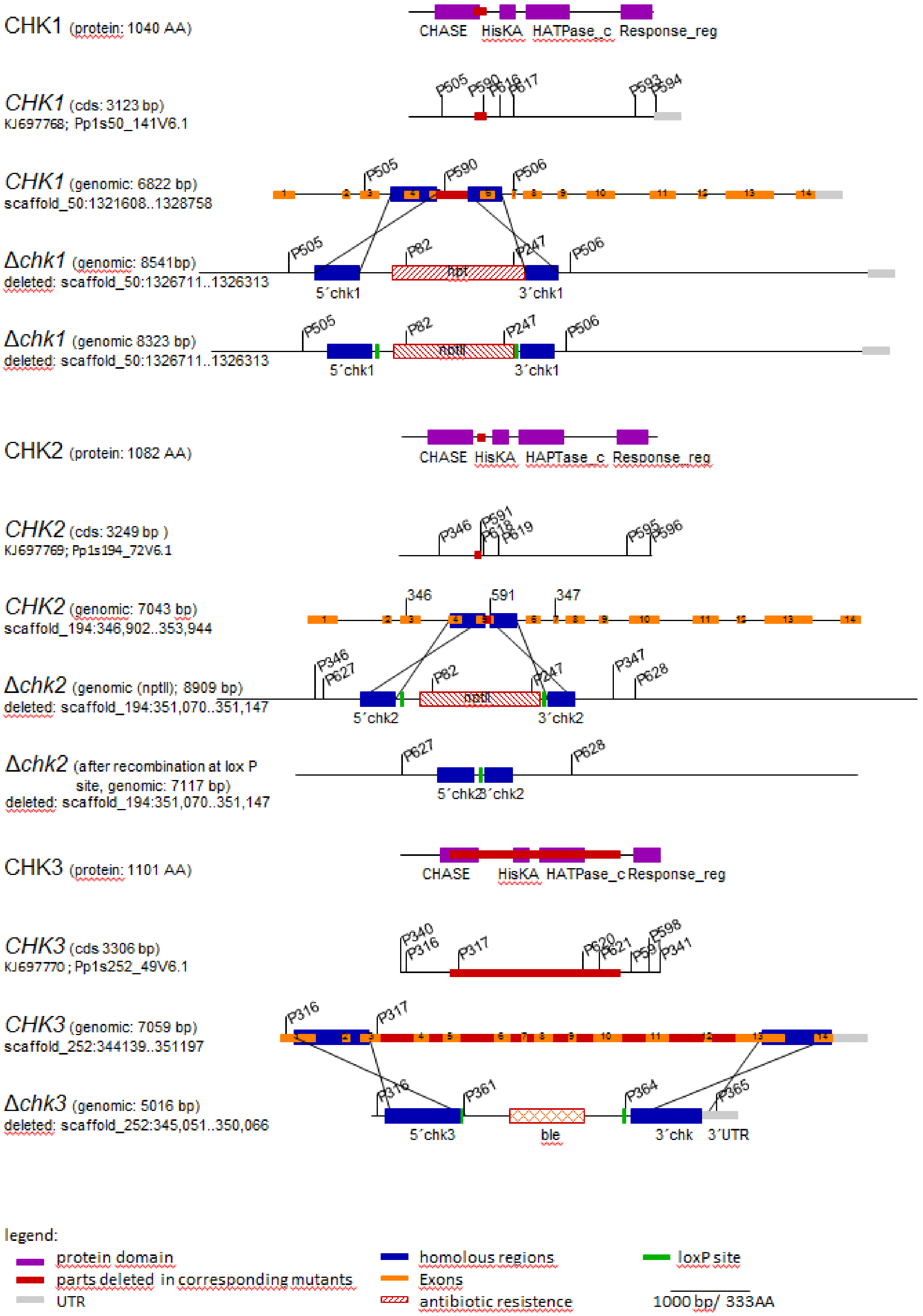

Fig. S 2. Overview of the *CHK* genes, proteins and knockout plants generated by transformation with the vectors listed in Tab. S 1. Double and triple mutants were generated by sequential transformation. The protein domains are shown in purple. The primers used for PCR (Fig. S 3) and RT-PCR (Fig. S 4) screening, as well as Real-time PCR Assays (Fig. 7) are indicated and sequences can be found in Tab. S 2. The accession numbers, as well as the Cosmoss gene (locus) IDs (cosmoss.org) are shown. Further the deleted regions in the corresponding mutants are shown in red and the deleted fragments are given by their scaffold location.

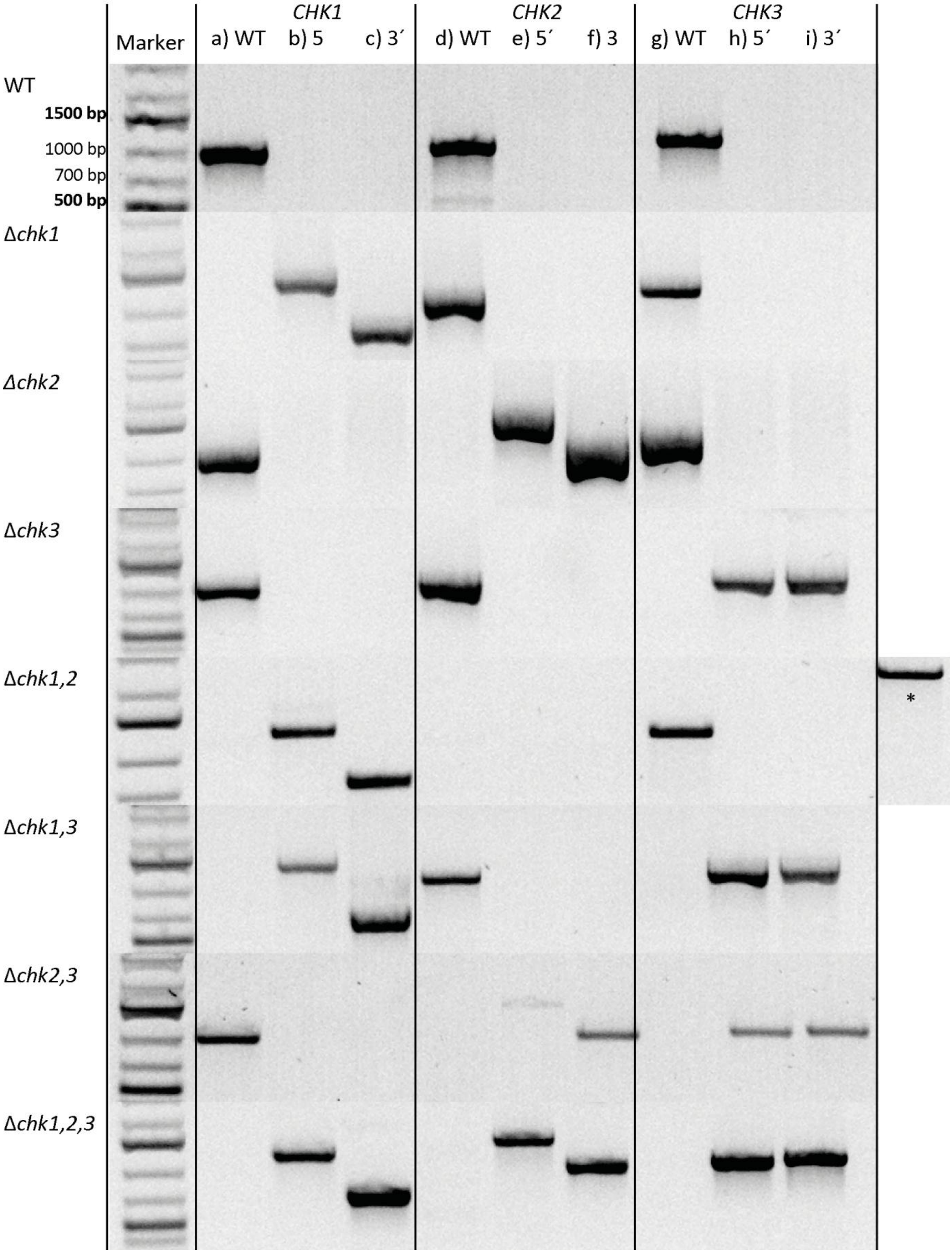

Fig. S 3. PCR screening of knockout mutants. DNA has been isolated (http://moss.nibb.ac.jp) from one colony of each stable mutant. For the WT and each mutant nine PCRs have been performed as described in Kamisugi and Cuming (2009, In Annual Plant Reviews: The Moss Physcomitrella patens, Vol 36. pp 76-112: For each locus forward and reverse primers were picked adjacent to the 5' homolog fragment of the respective locus (WT) to test the presence/absence of the WT locus. The same forward primer together with a reverse primer from the resistance cassette (5') as well as a forward primer from the resistance cassette together with a reverse adjacent to the 3' homolog fragment (3') were applied to show the correct integration at both sides after homologous recombination. *CHK1*: a) WT(P505/P590), b) 5´(P505/P82), c) 3´(P247/P506); d) *CHK2*: WT(P346/P591), e) 5´(P346/P82), f) 3´(P247/P347); g) *CHK3*: WT(P316/P317), h) 5´(P316/P361), i) 3´(P364/P365). * The nptII resistance cassette in the *Δchk1,2* mutants was removed from the *chk2* locus by transient CRE recombinase expression. Thus the products e and f are missing. Instead an additional PCR (P627/P628) was performed and the product was sequenced to confirm the deletion of a 77bp fragment. All primers are indicated in Fig. S 2 and are given in Tab. S 2.

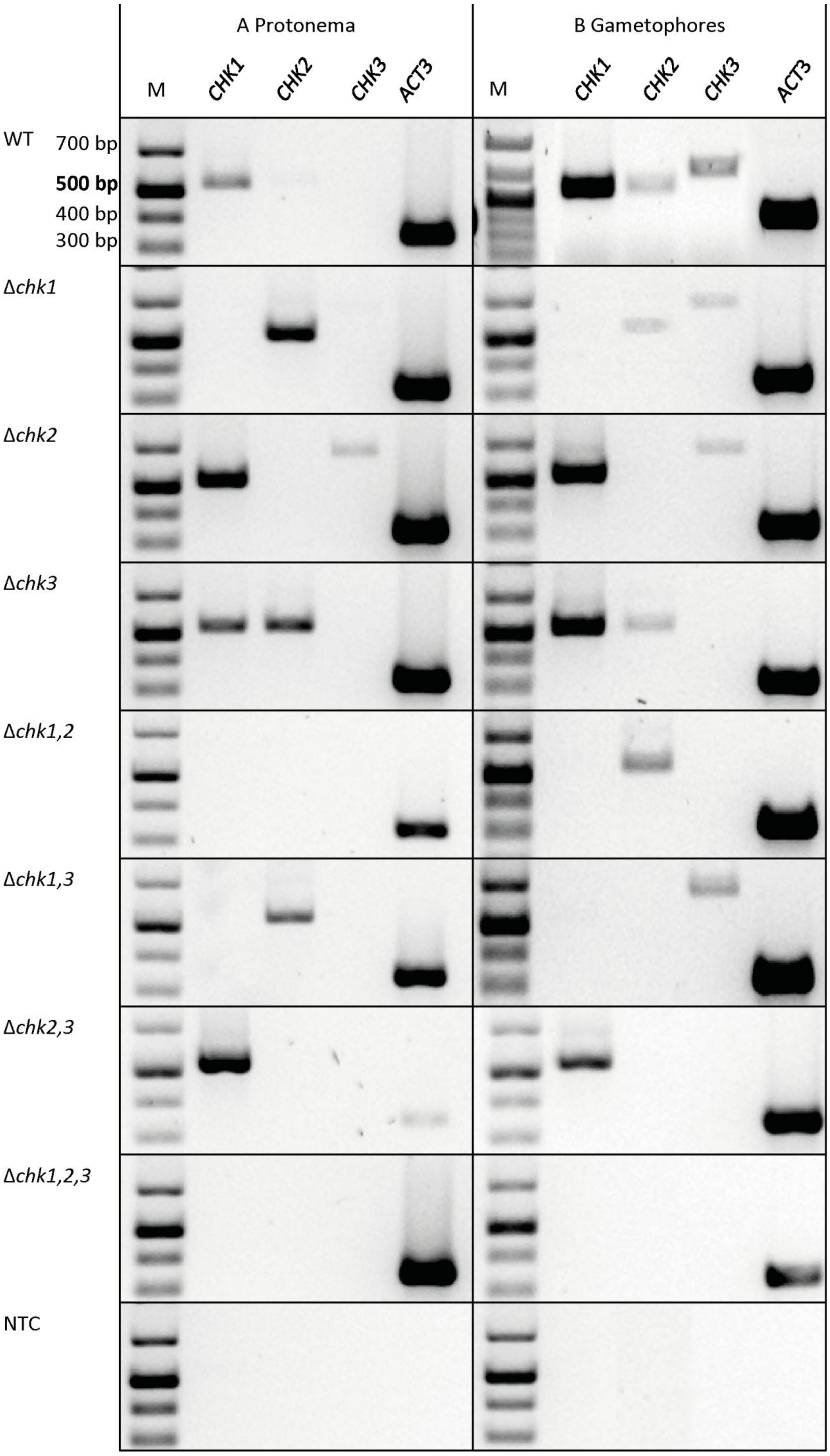

Fig. S 4. RT-PCR screen of the different mutant lines. RNA has been isolated from protonema (7-day-old) and gametophores (8-weeks-old) grown on solid medium in order to check the presence/abcence of the chk transcripts in the mutants. Two different stages of development were chosen here due to the fact that especially chk3 is on low abundance and thus not clearly detectable in protonema stages. RNA isolation and DNAse digestion has been performed as described in Material and Methods section. cDNA synthesis has been performed with RevertAid H Minus Reverse Transcriptase (Thermo Scientific). *CHK1* (P505/P590); *CHK2* (P346/P591); *CHK3* (P316/P317). NTC = non template control. All primers are indicated in Fig. S 2 and are given in Tab. S 2.

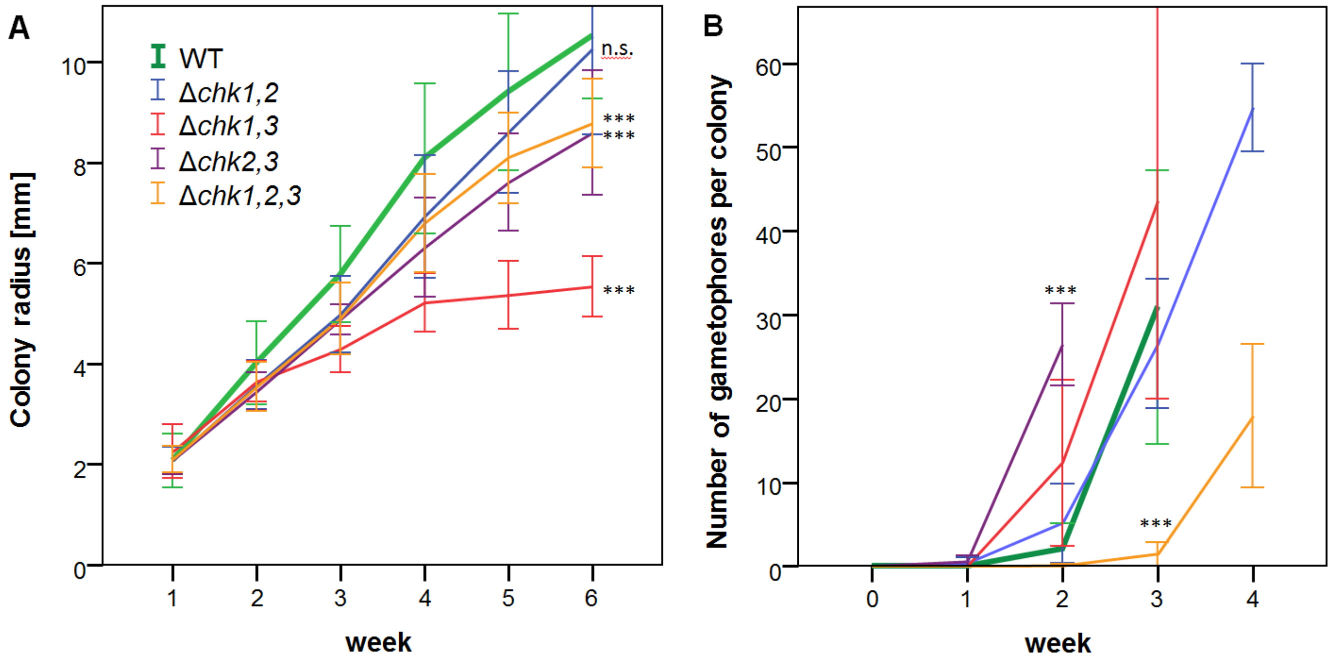

Fig. S 5. A) Average colony radius over the course of six weeks after inoculation of KNOP-Agar. B) Time course of gametophore frequency. Gametophores with at least three developed leaflets were counted up to 60 gametophores per colony (beyond that quantification was not further possible). Mean ± SD, t-test difference from WT, *** p<0.001, otherwise no significant differences.

Tab. S 1. Generation of *CHK* mutant collection.

Four different vectors were generated targeting the loci of *CHK1*, *-2*, and *-3* individually. They were designed using three different resistance cassettes in order to allow the generation of double and triple mutants by subsequent re-transformation of already characterized single and double mutants respectively. At least 330 bp of homologous region were cloned from genomic DNA on two sides of the resistance cassette aiming at targeted integration and subsequent disruption of one specific locus. The following characterization (antibiotic selection, PCR screen, RT/PCR screen and flow cytometry) were performed prior to re-transformation with another construct in order to guarantee ideal mutants. The nptII resistance cassette in the Δchk1,2 mutant was eliminated by site specific recombination using the cre/lox system by transient expression of Cre recombinase (Albert et al. 1995, Plant J 7: 649-659, Troulliet et al. 2006, Nucleic Acids Res 34: 232-242).

Vectors for generation of mutants of up to three different constructs. *pNPTIIRev and pNPTIIFor were obtained from Schaefer DG and Zryd JP (1997, Plant J 11: 1195-1206) and are a modified version of pHP23b. pHP23b was subcloned in the pBSII-KS plasmid (Stratagene) using *Eco*RI as restriction enzyme in reverse and forward orientation, leading respectively to the pNPTIIRev and pNPTIIFor plasmids. For pHygro, the NptII cassette of pNptIIFor was removed by *Eco*RI digestion and the hygromycin resistance marker gene from the pCambia vector (pC1304) was cloned at the blunted *Eco*RI site. **pBZR was obtained from Schaefer et al. (2010, DNA Repair 9: 526-533) and is a modified version of pBNR. The *Eco*RI/*Not*I fragment of pBNR, corresponding to the NPTII gene was replaced by an *Eco*RI/*No*t1 PCR amplified fragment of pLGZ2 (Kubo et al. (2013) Plos One 8: e77356) corresponding to the zeocin resistance gene leading to pBZR. The final alternations of the three different loci are shown in Fig. S 2.

Protoplastation and PEG mediated transformation using linearized Plasmid DNA was performed according to Schaefer et al. (1991, Mol Gen Genet 226: 418-424). Stable mutants were selected after three rounds of growth on solid medium with the corresponding antibiotic (G418 (50 µ µg/ml), Hygromycin B (30 µ µg/ml) and zeocin 30 µ µg/ml). The selected, stable mutants grow easily on antibiotic containing media whereas the WT and mutants other resistances die or grow poorly.

Protoplast fusion is known to occur during protoplastation and PEG transformation and results in various phenotypic alterations Schween et al. (2005, Bryologist 108: 27-35). All mutants have been checked using the PA Analyzer (Partec) for their relative genome size and are in haploid stage (data not shown).

**Mutant**	**Vector 1**	**Vector 2**	**Vector 3**	**Antibiotic-Resistence of mutants**
Δchk1	*pNPTIIRev_PpCHK1	-	-	Geneticin
Δchk2	*pNPTIIFor_PpCHK2	-	-	Geneticin
Δchk3	**pBZR_PpCHK3	-	-	Zeocin
Δchk1,2	pNPTIIFor_PpCHK2	*pHygro_PpCHK1	-	Hygromycin B<br>*(Geneticin resistance was eliminated by recombination)*
Δchk2,3	***pNPTIIFor_PpCHK2	pBZR_PpCHK3	-	Geneticin<br>Zeocin
Δchk1,3	pNPTIIRev_PpCHK1	pBZR_PpCHK3	-	Geneticin<br>Zeocin
Δchk1,2,3	pNPTIIFor_PpCHK2	pHygro_PpCHK1	pBZR_PpCHK3	Geneticin<br>Hygromycin B<br>Zeocin

Tab. S 2. Primer sequences. All Primers used in this study were obtained from Metabion (Planegg, Germany).

target/purpose	Primer	Sequence
nptII/hyg PCR screening	P82	actgtcggcagaggcatctt
	P247	gggtttcgctcatgtgttga
zeo cassette screening	P361	ggccggccagatctataac
	P364	cgaagttatctcgagtcgcg
CHK1 PCR screening	P505	catatgccgagagagtccttc
	P590	caaagtccaagtcgctgaca
	P506	atctcgtgagacacagttgcc
CHK2 PCR screening	P346	atttgctgaacgagtgctgc
	P591	gatctccgaaatccaagctg
	P347	gaatttcatgtgatactgtcgcc
	P627	gacgaatatgccccaaccac
	P628	acggcctgattcaatttttg
CHK3 PCR screening	P316	ggagtcagaagtgaagctagg
	P317	aaacggtctcttgagacattgta
	P365	atccaccttcacattcatgcg
*ACT*3	P214	cggagaggaagtacagtgtgtgga
	P215	accagccgttagaattgagcccag
Real-time 60S L21 Protein	P494	acgcaccggcatcgt
	P495	tgcttgttcatcacgacaccaa

Tab. S 3. Data for the budding assay (Fig. 5 & Fig. 6). Equal amounts of protonema were suspended on KNOP agar medium containing 0 - 400 nM iP as well as 400 nM *t*Z and BA and bud formation was analyzed microscopically after 10 days under standard conditions. The number of buds corresponds to one microscopic view field (3.8 mm^2). At least two different biological replicates were counted in 5 - 10 view fields and mean values were calculated, n = number of viewfields counted. t-test different from Wt at 400 nM, ** p< 0.01*** p<0.001, equal variances not assumed.

	CK	c [nM]	n	mean	SD	t-test
WT	iP	0	60	0	0	
		50	50	4.30	4.83	
		100	35	16.91	4.22	
		400	45	25.47	6.14	
	*t*Z	400	54	22.43	12.44	
	BA	400	80	19.25	10.81	
Δ*chk1*	iP	0	25	0	0	
		50	25	7.12	7.22	
		100	25	21.16	6.67	**
		400	27	15.11	10.60	***
	*t*Z	400	30	1.10	1.45	***
	BA	400	30	7.10	6.82	***
Δ*chk2*	iP	0	35	0	0	
		50	40	0.20	0.60	***
		100	30	2.50	2.46	***
		400	35	1.91	1.48	***
	*t*Z	400	40	0.68	1.33	***
	BA	400	20	1.30	0.84	
Δ*chk3*	iP	0	35	0	0	
		50	40	2.73	2.38	*
		100	35	10.97	7.57	***
		400	35	18.69	10.95	**
	*t*Z	400	30	5.67	3.75	***
	BA	400	30	7.07	4.02	***
Δ*chk1,2*	iP	0	30	0	0	
		50	35	0	0	***
		100	35	0.91	1.98	***
		400	35	1.51	2.02	***
	*t*Z	400	35	0.26	0.69	***
	BA	400	40	1.05	1.28	***
Δ*chk1,3*	iP	0	30	0	0	
		50	25	0.64	1.38	***
		100	15	16.33	10.10	n.s.
		400	30	17.47	8.91	***
	*t*Z	400	40	0.33	0.57	***
	BA	400	25	1.04	1.48	***
Δ*chk2,3*	iP	0	35	0.00	0.00	
		50	40	5.60	3.33	n.s.
		100	20	12.15	4.50	**
		400	25	9.44	7.96	***
	*t*Z	400	20	14.75	4.23	***
	BA	400	30	18.97	4.56	n.s.
Δ*chk1,2,3*	iP	0	20	0	0	
		50	20	0	0	***
		100	20	0	0	***
		400	20	0	0	***
	*t*Z	400	20	0	0	***
	BA	400	20	0	0	***
